# Beyond Critical Period Learning: Striatal FoxP2 Affects the Active Maintenance of Learned Vocalizations in Adulthood

**DOI:** 10.1523/ENEURO.0071-19.2019

**Published:** 2019-04-15

**Authors:** Nancy F. Day, Taylor G. Hobbs, Jonathan B. Heston, Stephanie A. White

**Affiliations:** Department of Integrative Biology and Physiology, University of California Los Angeles, Los Angeles, CA 90095

**Keywords:** auditory feedback, basal ganglia, birdsong, sensorimotor, speech

## Abstract

In humans, mutations in the transcription factor forkhead box P2 (FOXP2) result in language disorders associated with altered striatal structure. Like speech, birdsong is learned through social interactions during maturational critical periods, and it relies on auditory feedback during initial learning and on-going maintenance. Hearing loss causes learned vocalizations to deteriorate in adult humans and songbirds. In the adult songbird brain, most FoxP2-enriched regions (e.g., cortex, thalamus) show a static expression level, but in the striatal song control nucleus, area X, FoxP2 is regulated by singing and social context: when juveniles and adults sing alone, its levels drop, and songs are more variable. When males sing to females, FoxP2 levels remain high, and songs are relatively stable: this “on-line” regulation implicates FoxP2 in ongoing vocal processes, but its role in the auditory-based maintenance of learned vocalization has not been examined. To test this, we overexpressed FoxP2 in both hearing and deafened adult zebra finches and assessed effects on song sung alone versus songs directed to females. In intact birds singing alone, no changes were detected between songs of males expressing FoxP2 or a GFP construct in area X, consistent with the marked stability of mature song in this species. In contrast, songs of males overexpressing FoxP2 became more variable and were less preferable to females, unlike responses to songs of GFP-expressing control males. In deafened birds, song deteriorated more rapidly following FoxP2 overexpression relative to GFP controls. Together, these experiments suggest that behavior-driven FoxP2 expression and auditory feedback interact to precisely maintain learned vocalizations.

## Significance Statement

Mutations within the forkhead box P2 (FOXP2) gene impair speech and language. In zebra finch songbirds, the predominant model for investigating the neural and genetic mechanisms underlying human speech, FoxP2 is critical for song learning. Striatal FoxP2 expression levels correlate with song variability. We overexpressed FoxP2 in the striatopallidum of adult male zebra finches to assess its contribution to the maintenance of adult vocalizations independent of developmental perturbations. We tested the hypothesis that high FoxP2 expression promotes song stability by longitudinally assessing song in the presence and absence of auditory feedback and in two social contexts. We found that dysregulated FoxP2 interferes with hearing-dependent song maintenance. These results suggest that auditory-based regulation of FoxP2 is critical for the ongoing maintenance of adult vocalizations.

## Introduction

A foundation for humans’ ability to acquire language is speech, a learned vocal behavior that relies on sensorimotor experience. The discovery of a point mutation in the DNA binding domain of the forkhead box P2 (FOXP2) transcription factor in a British family with an inherited language impairment provided the first definitive link between this gene and speech and language (Lai et al., 2001). Individuals who inherit this mutation have speech deficits and structural abnormalities in the striatum, among other brain areas (Watkins et al., 2002).

The zebra finch songbird (*Taeniopygia guttata*), a species in which only males sing, is an essential animal model for studying learned vocal communication (Brainard and Doupe, 2013). Zebra finch song and human speech exhibit many parallels (Doupe and Kuhl, 1999), including (1) acquisition of species-specific acoustic signals (e.g., native language/tutor song) during a sensory critical period; and (2) refinement of immature vocal signals (e.g., babbling/subsong) into precisely-controlled, mature vocalizations (e.g., words/crystallized song) using auditory-guided learning during a sensorimotor critical period (Bolhuis et al., 2010; Brainard and Doupe, 2013). Vocal plasticity persists into adulthood such that both groups are able to continually modify their vocalizations to maintain appropriate vocal output (Tumer and Brainard, 2007; Andalman and Fee, 2009; Sober and Brainard, 2009). However, in the absence of auditory feedback, vocalizations slowly deteriorate (Konishi, 1965; Cowie et al., 1982; Nordeen and Nordeen, 1992).

Fortuitously in songbirds, the neural circuitry that supports vocal learning, production and maintenance is composed of discrete, interconnected, and song-dedicated nuclei. One group of nuclei is critical for song production, and a cortico-basal ganglia-thalamo-cortical loop (the anterior forebrain pathway; AFP) is necessary for song learning. Within area X, a nucleus that contains striatal and pallidal cell types (Farries and Perkel, 2002), *FoxP2* is dynamically regulated both by singing and the social context in which song is sung, as follows, In adults, expression is reduced following 2 h of singing alone (undirected song; UD) relative to the robust levels observed following 2 h of female-directed singing (FD; male courting a female) or in males that do not sing. In both adults and juveniles, the more the male sings alone, the lower its FoxP2 levels (Teramitsu and White, 2006; Miller et al., 2008; Teramitsu et al., 2010; Hilliard et al., 2012b; Chen et al., 2013; Shi et al., 2013; Thompson et al., 2013). Interestingly, when juvenile birds are deafened, singing-driven downregulation of *FoxP2* is no longer correlated with how much the bird sings (Teramitsu et al., 2010), suggesting that *FoxP2* levels are calibrated by auditory feedback to guide sensorimotor learning.

Interfering with behavior-linked *FoxP2* levels using viral-mediated knock-down or overexpression interferes with juvenile song learning such that birds are unable to properly imitate their memorized auditory template (Haesler et al., 2007; Heston and White, 2015; Burkett et al., 2018). Together, these data indicate that behavior-linked regulation of FoxP2 is critical for song development as young birds engage in trial-and-error learning to adaptively sculpt their vocalizations. In adults, knock-down of *FoxP2* prevents social context-dependent alterations to song (Murugan et al., 2013), suggesting that inappropriate *FoxP2* expression also impairs the precision of crystallized song.

To reveal whether FoxP2 participates in active song maintenance, we prevented behavior-driven downregulation of FoxP2 by overexpressing FoxP2 in area X of adult male zebra finches and deafened a subset of them, similar to manipulations that demonstrated a key role for the AFP in adult song plasticity (Brainard and Doupe, 2000). A simple prediction was that high FoxP2 levels would promote song stereotypy, as is observed following performance of FD song or singing quiescence. However, we observed that constitutively high FoxP2 accelerated song deterioration in deafened birds. We also analyzed song produced in two social contexts (UD and FD), and conducted female preference tests to determine if the resultant high vocal variability in FD song was behaviorally-meaningful.

## Materials and Methods

### Subjects

All animal use was in accordance with NIH guidelines for experiments involving vertebrate animals, approved by the University of California Los Angeles Chancellor’s Institutional Animal Care and Use Committee, and consistent with the American Veterinary Medical Association guidelines. Birds from our breeding colony were housed in climate-controlled rooms inside of cages and/or aviaries. A 14/10 lights on/lights off cycle was maintained; 30 min of dawn and dusk lighting was simulated each morning and evening. Birds had unlimited access to food, grit, and water, and were provided nutritional supplements (e.g., spray millet, green vegetables, and calcium supplements) and environmental enrichments (e.g., a variety of perches, swings, mirrors and water baths).

### Experimental timeline

Twenty-five male zebra finches [>120 d post hatch (dph), mean age = 153 dph] were recorded in sound attenuation chambers for a minimum of two weeks (PRE) before injection of adeno-associated virus (AAV), serotype 1 (AAV1), driving expression of zebra finch FoxP2 or of GFP (surgery A; FoxP2-AAV = 13, GFP-AAV = 12, mean age = 178 dph). We used AAV constructs previously described (Heston and White, 2015; Burkett et al., 2018), and followed those surgical procedures with the exception that 500 nl of virus was injected per hemisphere.

**Figure 1.**
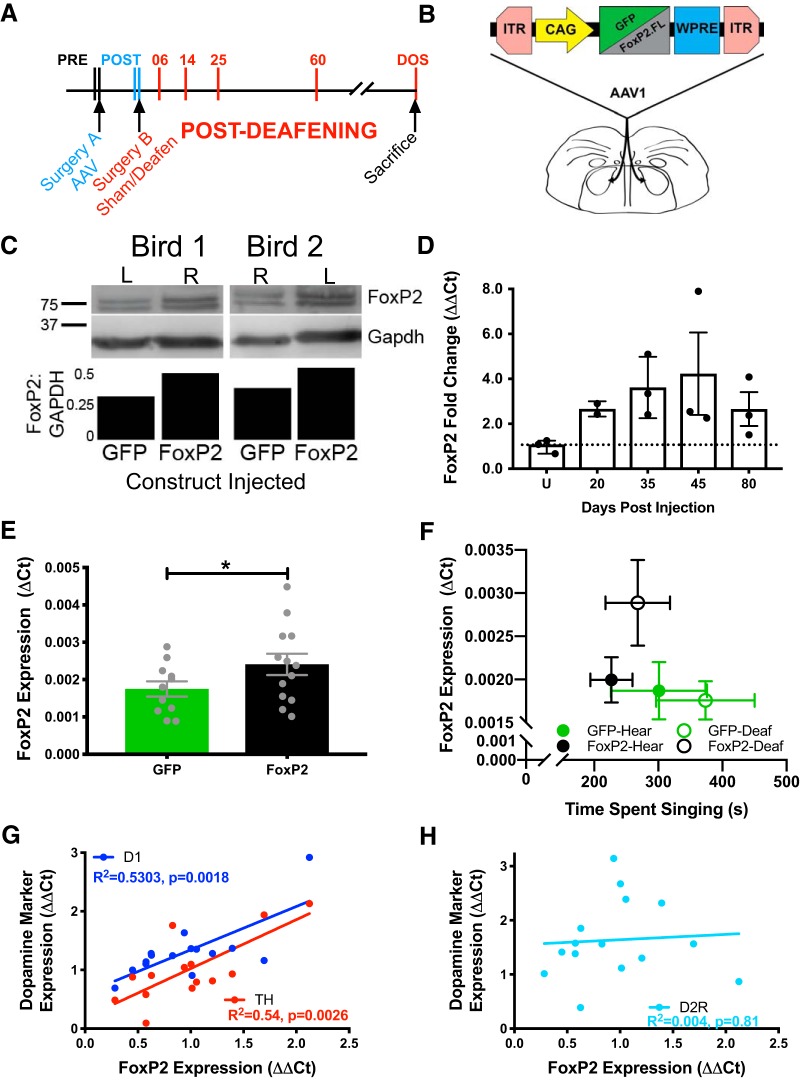
AAV construct drives overexpression of FoxP2 in adult male zebra finches. ***A***, Timeline of experimental manipulations. The song-dedicated striatal nucleus area X was bilaterally injected with an AAV construct (surgery A) to drive overexpression of GFP (control) or FoxP2. To remove auditory feedback in half of the birds, surgery B was performed ∼20 d following surgery A. Songs were analyzed (vertical lines) at two time points directly before each surgery and at 6, 14, 25, and 60 d after deafening (e.g., D06, D14, etc.), and on the morning of sacrifice (DOS). ***B***, Schematic of the AAV construct used to drive expression either GFP or FoxP2 using the CAG promoter. ***C***, Protein levels of FoxP2 appear higher in hemispheres injected with FoxP2 compared to hemispheres injected with GFP in the same bird. ***D***, In hearing birds used for evaluating the time line of *FoxP2* overexpression, RT-qPCR confirms augmented levels at 20, 35, 45, and 80 d after injection (equivalent to surgery A time point in panel ***A***) relative to uninjected controls (U). Fold change values are normalized to the mean of the controls. ***E***, Across all birds used for behavioral analysis, *FoxP2* expression levels (ΔCt; mean ± SEM) are higher in FoxP2-injected versus GFP-injected birds (*p* = 0.042), on the morning of sacrifice (DOS) approximately six months after surgery A. ***F***, ΔCt values of *FoxP2* levels on DOS and time spent singing for each group shows that FoxP2-Deaf birds trend toward higher FoxP2 expression despite singing similar amounts as other groups (mean ± SEM). ***G***, ΔΔCt values of FoxP2 levels positively correlate with dopamine markers D1R and TH, but not with D2R (***H***). In ***D–H***, dots represent individual birds. *Figure Contributions:* Jon Heston identified the appropriate viral construct. Nancy Day performed the experiments and analyzed the data. *p < 0.05.

At ∼30 d following viral injection (range: 21–40 d), birds were re-recorded for 2 d (POST). All birds were then subjected to a second surgery (surgery B, mean age = 208 dph), in which half of the birds were deafened via cochlear extraction (*n* = 12) and half were sham-deafened (*n* = 13) as described by Teramitsu et al. (2010). Birds were intermittently recorded for the following five months; songs were analyzed at 6, 14, 25, and 60 d (D06, D14, D25, D60) after deafening, and on the day of sacrifice (DOS). Time points were chosen to coincide with when changes to songs might be expected based on prior studies (e.g., D06; Horita et al., 2008). Birds were sacrificed ∼185 d following AAV injection (min = 182 d, max = 200 d). Birds were sacrificed by decapitation following 2 h of UD singing, and brains were rapidly extracted and frozen by liquid nitrogen. A timeline for these experiments in schematized in Figure 1*A*.

### Overexpression validation

Verification of targeting and overexpression of zebra finch *FoxP2* mRNA for all birds in which behavior was analyzed was done using *in situ* hybridization (data not shown) as described by Teramitsu and White (2006) and by RT-qPCR on tissue punches as described by Burkett et al. (2018). *FoxP2* expression was quantified relative to *Gapdh* (ΔCt).

To specifically assess FoxP2 protein levels following viral injection, two adult males were each injected with 500-nl FoxP2-AAV in area X of one hemisphere, and with 500-nl GFP-AAV in the other. This approach allowed us to control for any difference in FoxP2 levels that are a result of dynamic behavioral regulation or inter-bird differences. After three weeks, males sang alone for 2 h in the morning and were then sacrificed by rapid decapitation. Brains were extracted, flash frozen on liquid nitrogen and cryosectioned (Leica Microsystems) in the coronal plane at a thickness of 30 µm. Tissue punches of area X were made using a 20-gauge Luer adapter (BD) at a depth of 1 mm as in Miller et al. (2008). Western blotting was also as described in Miller et al. (2008). Expression levels of FoxP2 in Figure 1*C* are presented and quantified as percentage change in the AAV-FoxP2 hemisphere relative to the AAV-GFP hemisphere.

A second group of males (*n* = 15, mean age = 163 dph) was used to verify persistent overexpression of FoxP2 across the experimental time course and to coincide with the time points in which song behavior was analyzed (e.g., Fig. 1*A* experimental time course D14 post-deafening corresponds to Fig. 1*D* post-injection day 35 in the AAV expression time course validation). Of these, 12 birds received 500 nl of AAV-FoxP2 to each area X after which three were sacrificed at each time point (20, 35, 45, and 80 d post-surgery); three birds (180 dph) served as uninjected controls. At each time point, birds were rapidly decapitated in the morning before any song had been produced, and brains were extracted, frozen on liquid nitrogen, and stored at –80°C until use. Tissue punches from area X and the adjacent ventrostriatal pallidum (VSP) were homogenized in 100 µl Qiazol (QIAGEN) and total RNA was extracted using the Direct-Zol MicroRNA Prep kit (Zymo Research). A total of 100-ng RNA was reverse-transcribed to cDNA (Applied Biosystems High Capacity RNA-to-cDNA kit, #4387406) for qPCR (as described above). The ΔΔCt method (Livak and Schmittgen, 2001) was used to calculate fold-changes in the expression FoxP2, the D1 and D2 dopamine receptors (D1R and D2R, respectively), as well as the dopamine biosynthetic enzyme tyrosine hydroxylase (TH), relative to Gapdh in area X compared to VSP. Primer sequences were designed for zebra finch D1R (112 bp), D2R (206 bp), and TH (107 bp) using the NCBI Primer Design Tool, and were validated using melt curve analysis and standard curves. Primers sequences were: D1R FOR: CCGGGAGGACATTACAGTTTAG; D1R REV: TGCAGTTCCACCCGTATTTAG; D2R FOR: CCCAGCAGAAGGAGAAGAAAG; D2R REV: CTCGATGTTGAAGGTGGTGTAG; TH FOR GCACCCTGAAGAGCTTGTAT; TH REV: CAGCTGAGGGATGTTGTTCT.

### Song recording and analysis

UD song was collected across the entirety of the experiment by housing animals singly within a sound attenuation chamber. Although animals were moved to social housing in between experimental time points, each bird was recorded within the same isolation chamber for the duration of the experiment. All reasonable attempts were made to record a given bird using the same microphone and recording devices/settings, with occasional differences in the quality of recordings between time points. Sounds were acquired using Shure SM58 microphones, digitized using a PreSonus Firepod or AudioBox (44.1-kHz sampling rate, 24-bit depth) and recorded using Sound Analysis Pro (SAP) 2011 software (Tchernichovski et al., 2000).

Songs were analyzed at the level of the motif as well as the syllable, each of which were hand-segmented using custom-written MATLAB code (Tumer and Brainard, 2007). Motifs were identified as repeated units of song composed of multiple syllables. Introductory notes were included in all analyses to assess any effect of stuttering following deafening (Horita et al., 2008; Kubikova et al., 2014). Canonical and non-canonical renditions of motifs were included in the analyses to capture the full range of singing behavior. A syllable was identified as a sound element that is separated from other syllables by silence or by local minima in the amplitude. Motif similarity as well as the phonology and syntax of syllables were compared to PRE vocalizations at each subsequent time point (Fig. 1*A*), as specified below.

#### Motif similarity

The similarity index (Mandelblat-Cerf and Fee, 2014) quantified how well birds imitated their PRE motifs. Twenty motifs, collected from songs produced on two consecutive mornings, that were sung within one week before surgery A (PRE) were compared against 30 song bouts for each day included in the analysis (PRE1, PRE2: morning of surgery A; POST1, POST2: morning of deafening, D06, D14, D25, D60, DOS). Of note, PRE1 and POST1 were dates immediately preceding PRE2 and POST2.

#### Syllable similarity

The first ∼450 syllables of each analysis time point were segmented within MATLAB using an amplitude threshold, grouped into syllable clusters, and assigned an arbitrary label using the semi-automated clustering algorithm VoICE (Burkett et al., 2015). All spectral features were calculated using sound analysis tools (SAT; http://soundanalysispro.com/matlab-sat) in MATLAB. We quantified both syllable similarity to PRE using custom-written MATLAB code derived from the similarity batch function of SAP 2011 (Tchernichovski et al., 2000; Burkett et al., 2015). To calculate syllable similarity over time, 30 renditions of each syllable at each time point were compared to 30 renditions of that syllable produced during PRE. Syllable similarity was represented by the mean of these 900 comparisons, and normalized to the mean of PRE versus PRE1 and PRE versus PRE2 to account for day-to-day variability within a bird. Higher scores indicate greater similar to songs produced before viral (surgery A) or auditory manipulation (surgery B).

#### Spectral variability

For each bird and time point, the coefficient of variation (CV) was calculated using the first 40 renditions of each syllable for the following acoustic features: entropy, entropy variance, duration, pitch goodness, pitch, and frequency modulation (FM). All acoustic features were calculated using SAT. To assess how these syllables changed relative PRE, the mean CV effect size (CV ES) for each bird was calculated by averaging the CV ES of all syllables. The CV ES for each syllable was determined using the following formula: CV ES = (CV_Time Point_ – CV_Pre_)/(CV_Time Point_ + CV_Pre_).

#### Syllable preservation

We calculated both the number of syllables that remained in a bird’s motif and the number of syllables that were added to a bird’s motif following deafening. First, the “core syllables” of a motif were identified as syllables that were present in >60% of a bird’s motifs before deafening. An average syllable preservation percentage was calculated by dividing the total number of core syllables present each day by the total syllables produced on that day. For example, a syllable preservation score of 0.95 indicates that 95% of the syllables produced that day were syllables integral to a motif.

#### Syntax analysis

For each bird and time point, we created a transition probability matrix from strings of identified syllables. Transition probability matrices of PRE versus each time point were correlated and included syllables that were omitted or introduced following deafening. A similarity score of 0 reflects no relationship to PRE sequencing, whereas a score of 1 indicates an exact match to PRE sequencing (Miller et al., 2010; Burkett et al., 2015).

#### Social context

We elicited FD song from male birds (*n* = 13 birds, *n* = 23 syllables) before and following viral overexpression of GFP or FoxP2. A rotation of six female zebra finches was used to prompt courtship song over the course of 2 h. Females were placed in the cage with the male for 10 min at a time, removed, and replaced with another female. All interactions were video recorded and visually monitored to verify that males were directing their songs to a female. To assess variability in pitch, the fundamental frequency (FF) was measured for syllables containing harmonic elements in both UD and FD song epochs. The CV of the FF was calculated using 25 pseudorandomly-selected renditions of each syllable in each context. Syllables that did not exhibit the characteristic decrease in CV_FF_ during FD song (Kao and Brainard, 2006) in the PRE condition were excluded from all analyses (*n* = 8).

### Female preference

To determine whether FoxP2 overexpression influenced courtship song quality, sexually-naive females were used to assess preference for songs produced before and after viral injection. Mature female finches (*n* = 35; 100–120 dph) were selected from female-only group housing and moved to individual cages within sound attenuation chambers. Cages (38 × 25 × 28 cm) were outfitted with two static perches and two “switch” perches that lowered when the bird landed on them. Switch perches were made by securing a 6-cm red pipe cleaner to a miniature switch requiring minimal force (Cherry D429-R1ML) and were placed on the back wall of the cage, each 4 cm from the side walls and 15 cm from the ground. A vertical plastic barrier (12 cm) was placed in the middle of the cage to create separate, but connected, areas of the cage, and to impede spurious motion from one side to the other. A single speaker (Pioneer Electronics) was placed behind the barrier. Activation of a switch resulted in sound playbacks. Playbacks were controlled using the “operant conditioning” module of SAP 2011 with a NI USB6501 (National Instruments).

#### Stimuli

Playbacks consisted of sound files containing two to five motifs. Five representative song files were generated for each of the four social contexts (UDPre, UDPost, FDPre, FDPost) and were selected for playback in a random order by SAP2011. All songs were unfamiliar to females; none had interacted with any of the males whose song was presented during the trials. Females were trained to associate perch activations with sound playbacks using Isolate song and FD song. “Isolate song” is produced by birds raised in the absence of a tutor and is not preferred by females (White, 2001).

#### Preference testing

For each trial, females participated in two phases of testing: “silence” and “playback.” During the silence phase (2 h), beginning at lights on, we determined a perch bias (PP, preferred perch; UP, nonpreferred perch) by observing the number of activations on each of the perches in the absence of auditory stimuli. FD song was always paired with the perch that received fewer perch activations to counteract the perch bias. Females (*n* = 16) that were unable to overcome their perch preference to demonstrate a song preference for FD song were excluded from further testing. A trial was excluded from analysis if the female failed to activate each perch five times during each of the silence and testing phases. Each male was tested by a minimum of five females who were tested on both PRE and POST songs. Song sets were grouped relative to surgery A, such that females only heard PRE or POST songs in a given trial (e.g., UDPre vs FDPre). Females were tested a minimum of three times on each set of songs (min = 3, max = 6, average = 3.5). A preference score, taking into account the perch bias during the silence phase, was calculated using the following formula:

$$\text{preference score =}\frac{\left[{\mathrm{Playback}}_{FD}-{\mathrm{Playback}}_{UD}\right]}{\left[{\mathrm{Playback}}_{FD}+{\mathrm{Playback}}_{UD}\right]}-\frac{\left[{\mathrm{Silence}}_{UP}-{\mathrm{Silence}}_{PP}\right]}{\left[{\mathrm{Silence}}_{UP}+{\mathrm{Silence}}_{PP}\right]}$$

A preference score > 0 indicates preference for FD song; negative values indicate a preference for UD song.

### Experimental design and statistical analysis

The criterion for statistical significance was set at α = 0.05. All significance levels were calculated as two-tailed except for cases in which we had prior experimental expectation of the outcome. Such cases are noted the text. Prism 8 (GraphPad) was used to perform all statistical tests. A D’Agostino and Pearson normality test was performed on each data set to determine normality. To calculate statistically-significant effects over time, metrics from each time point were compared to PRE using a Kruskal–Wallis one-way ANOVA within each of the four groups (e.g., FoxP2-Hear, GFP-Deaf). Details for all statistical tests are included in either the results or figure legends.

### Code accessibility

Custom-written MATLAB code (NFD) for the generation of syllable similarity scores using the Similarity Module is adapted from Burkett et al. (2015).

## Results

### Overexpression of FoxP2 in area X of adult zebra finch males

AAV1 and the CAG promoter were used to drive overexpression of FoxP2 or GFP (Fig. 1*B*) in the song dedicated striatal nucleus, area X, in adult zebra finch males. This viral construct has been previously used to elevate FoxP2 levels in area X of young songbirds, which resulted in vocal learning deficits (Heston and White, 2015; Burkett et al., 2018). To validate expression in adults, first, Western blot analysis of protein from two birds demonstrated that within each bird, FoxP2 was elevated in area X of the hemisphere injected with AAV-FoxP2 relative to that injected with AAV-GFP (Fig. 1*C*; see Materials and Methods). Second, *FoxP2* mRNA was quantified using *in situ* hybridization (data not shown; see Burkett et al., 2018) and qRT-PCR as follows: In the cohort of unrecorded birds that were used to assess the time course of FoxP2 overexpression, high area X FoxP2 levels persisted in all animals for ≥80 d following injection compared to age-matched uninjected animals (Mann–Whitney *p* = 0.0002, one-tailed; uninjected *n* = 3 vs injected *n* = 11; Fig. 1*D*). To improve the clarity of Figure 1*D*, data from one bird in the 20-d group that received AAV-FoxP2 was removed for having FoxP2 expression 2 SD greater than the mean (ΔCt = 8.74, mean with bird = 3.65, mean without bird = 1.11). Inclusion of that data point would not alter the direction of the reported changes. Importantly, these animals were sacrificed without having sung, as FoxP2 mRNA and protein levels vary depending on how much a bird sings (Teramitsu and White, 2006; Miller et al., 2008; Teramitsu et al., 2010). Among the birds whose behavior was analyzed for this study and who were permitted to sing for 2 h before sacrifice, FoxP2-injected animals showed an increase in *FoxP2* expression compared to GFP-injected animals (*p* = 0.04; one-tailed unpaired *t* test, FoxP2 *n* = 13; GFP *n* = 11; Fig. 1*E*). Interestingly, separation of these two groups (FoxP2-injected and GFP-injected) into hearing and deaf subgroups suggests that this increase is largely driven by the FoxP2-deafened animals (Fig. 1*F*). This trend toward an increase in the FoxP2-deaf animals (one-way ANOVA: *F*_(3,20)_ = 2.14, *p* = 0.127) is not due to less singing as the average time spent singing did not differ among the four groups (mean, seconds ± SEM: FoxP2-Hear – 226.8 ± 32.8; FoxP2-Deaf – 268.0 ± 50.4; GFP-Hear – 301.0 ± 75.0; GFP-Deaf – 373.5 ± 76.9; one-way ANOVA: *F*_(3,17)_ = 1.115, *p* = 0.370).

### FoxP2 overexpression positively correlates with dopaminergic markers D1R and TH

To further validate our viral manipulation, we predicted that overexpression of FoxP2 would change the expression of specific markers in area X. Prior work shows that knocking down FoxP2 in area X leads to diminished expression of certain dopamine markers, including D1R (Murugan et al., 2013). We found that *D1R* (Spearman’s *r* = 0.62; *p* = 0.016, *n* = 15 pairs) and *TH* (Spearman’s *r* = 0.60; *p* = 0.026, *n* = 15 pairs) were positively correlated with FoxP2 expression (Fig. 1*G*). *D2R* expression levels were not correlated with *FoxP2* expression (Spearman’s *r* = 0.153, *p* = 0.58, *n* = 15 pairs; Fig. 1*H*), consistent with a previous study that identifies co-localization of Foxp2 with D1R, but not D2R in mouse striatum (Fong et al., 2018).

### UD quality and sequencing is unaffected by FoxP2 overexpression in hearing adults

**Figure 2.**
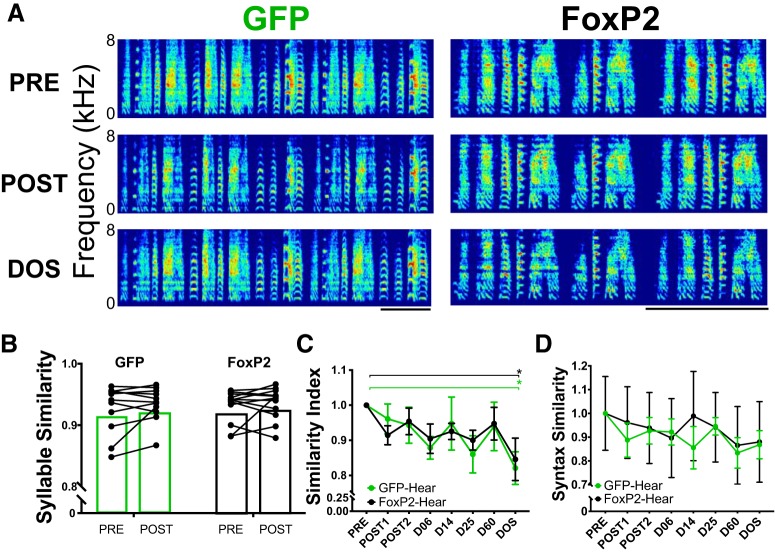
In hearing adults, area X FoxP2 overexpression does not alter UDs relative to those of GFP control birds. ***A***, Representative spectrograms of song bouts from two zebra finches before and after injection of an AAV that drives expression of a control GFP or FoxP2 construct. Scale bars = 500 ms. ***B***, Mean syllable identity is unchanged following FoxP2 overexpression. ***C***, Following surgery, UDs were similar to pre-surgery songs (PRE) except at the final six-month time point (DOS) for both GFP-injected and FoxP2-injected birds; *p < 0.05. ***D***, Syllable sequence (syntax similarity) is not altered following injection of AAV-GFP or AAV-FoxP2. *Figure Contributions:* Nancy Day performed the experiments and analyzed the data.

Overexpression or knock-down of FoxP2 in area X during sensorimotor learning impairs vocal learning (Haesler et al., 2007; Heston and White, 2015; Burkett et al., 2018). However, no role for FoxP2 in the maintenance of adult vocalizations, such as crystallized song, has been described. Overall, the songs produced following AAV-FoxP2 were visually similar to songs sung before surgery (Fig. 2*A*,*B*). To check for any subtle alterations to song, we examined syllable and motif similarity produced 3 weeks after surgery (POST1, POST2) to syllables and motifs produced before surgery (PRE). As a proxy for syllable “quality,” syllable similarity scores were calculated using MATLAB code (Burkett et al., 2015). A set of PRE syllables from 2 d just before surgery was compared against a set of syllables produced the morning before AAV injection. POST syllables from two consecutive days >20 d following surgery were combined to compare against the same set of PRE syllables. No differences in syllable similarity (AAV-GFP = 12 birds; AAV-FoxP2 = 13 birds) were detected for either group PRE versus POST (AAV-GFP: *p* = 0.278, two-tailed paired Wilcoxon; AAV-FoxP2: *p* = 0.677, two-tailed paired Wilcoxon; Fig. 2*B*).

Motif-level analyses were also performed to detect overall changes to song structure, including spectral quality and sequencing. The similarity index (Mandelblat-Cerf and Fee, 2014) was used as an unbiased metric to compare all songs performed following AAV injection and/or sham deafening surgeries to PRE song (Fig. 2*C*). Five PRE motifs were selected and scored against 20 bouts produced by each individual at each time point (POST1, POST2, and D06, D14, D25, and D60 after sham or deafening surgeries, and the DOS). A two-way ANOVA indicated a significant main effect of time, *F*_(7,78)_ = 3.15, MS = 0.033, *p* = 0.006. No significant main effect was detected for group (*F*_(1,78)_ = 0.057, MS = 0.0006, *p* = 0.815), nor for interaction between group and time (*F*_(7,78)_ = 0.230, MS = 0.002, *p* = 0.977). *Post hoc* analyses using Sidak’s multiple comparisons test showed that similarity scores at DOS for hearing birds were significantly different from PRE for both the AAV-GFP and AAV-FoxP2 groups (GFP: *p* = 0.023; FoxP2: *p* = 0.043); no other time points significantly differed from PRE.

Finally, we examined the sequencing of syllables using a weighted syntax score (Fig. 2*D*). As with overall similarity, we saw no differences between groups or within groups at any time point (two-way ANOVA: main effect for time, *F*_(7,79)_ = 1.60, MS = 0.029, *p* = 0.148; main effect for group, *F*_(1,79)_ = 0.373, MS = 0.007, *p* = 0.543; interaction, *F*_(7,79)_ = 0.159, MS = 0.003, *p* = 0.992). The variability of syntax scores in the AAV-FoxP2 group can be attributed to two animals whose syntax was variable from the onset of behavioral analysis (PRE-PRE comparisons were 0.49 and 0.46, compared to the other five animals in the group whose scores were all >0.90; all animals in the AAV-GFP group had >0.95 PRE similarity scores).

### FoxP2 overexpression hastens deafening-induced song deterioration

Crystallized zebra finch song is characterized by highly stereotyped sequences of syllables and low phonological variability. Given that we might not observe obvious differences in song following overexpression of FoxP2 due to the relative stability of the behavior, we deafened a subset of birds who received AAV-GFP and AAV-FoxP2 to eliminate auditory feedback, a manipulation that causes degradation of vocalizations (Nordeen and Nordeen, 1992; Woolley and Rubel, 1997). This manipulation allowed us to test whether or not FoxP2 overexpression alters deafening-induced song deterioration. Behavioral variability is correlated with singing-induced downregulation of FoxP2 juvenile finches (Miller et al., 2008), whereas highly-stereotyped FD is correlated with robust expression of *FoxP2* mRNA (Teramitsu and White, 2006). Thus, one hypothesis was that preventing FoxP2 downregulation would stabilize song, reducing its rendition-to-rendition variability, and delay song deterioration following the removal of auditory feedback. In contrast, we observed that deafening coupled with FoxP2 overexpression accelerated the deterioration of adult song.

**Figure 3.**
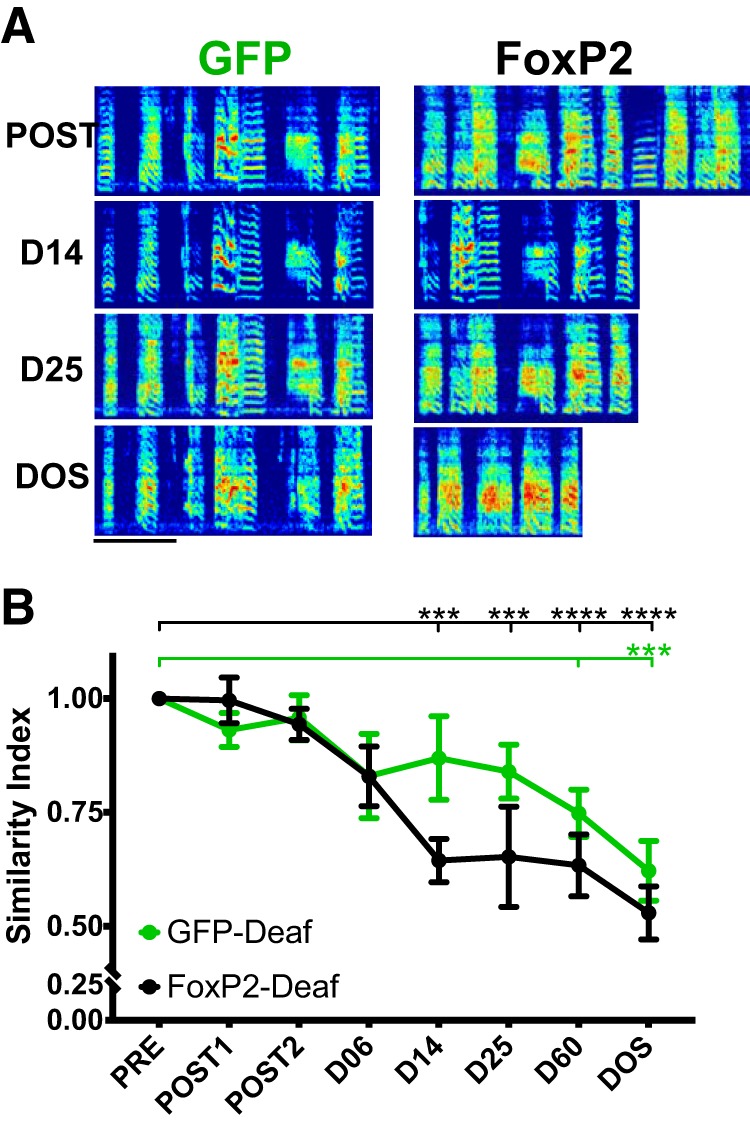
FoxP2 overexpression hastens deafening-induced song deterioration. ***A***, Representative spectrograms show deafening-induced song deterioration in two brothers who were deafened at 180 dph (surgery B) 29 d after injection of AAV-GFP (left) or AAV-FoxP2 (right; surgery A; Fig. 1). Scale bar = 500 ms. ***B***, At 14 d post-deafening, motif similarity is persistently altered in the AAV-FoxP2 group (two-way ANOVA with Sidak’s multiple comparisons, *p* = 0.0006, *p* = 0.0002, *p* < 0.0001, and *p* < 0.0001 at D14, 25, 60, and DOS, respectively, *n* = 6 birds). In comparison to AAV-GFP-deafened birds, degradation of songs by AAV-FoxP2-injected birds is accelerated by at least 10 d. Statistically significant changes to songs by AAV-GFP-deafened birds are present at DOS (two-way ANOVA with Sidak’s multiple comparisons, *p* = 0.0007, *n* = 5 birds). All motif similarity scores are normalized to motif similarity calculated between songs collected on 2 d before AAV injection (refer to Fig. 1*A*). ***p < 0.001, ****p < 0.0001. *Figure Contributions:* Nancy Day performed the experiments and analyzed the data.

Representative spectrograms from two deafened siblings show that the brother who received AAV-FoxP2 had more profound alterations to his song (Fig. 3*A*,*B*). To quantify this change, we performed motif/bout level similarity scoring to PRE song at four time points following deafening (D06, D14, D25, and D60) and on the DOS. A two-way ANOVA confirmed that both time (*F*_(7,70)_ = 11.64, MS = 0.246, p < 0.0001) and group (*F*_(1,70)_, MS = 0.102, *p* = 0.031) were significant main effects (interaction: *F*_(7,70)_ = 1.163, MS = 0.025, *p* = 0.335). Within the groups, compared to PRE, Sidak’s multiple comparisons tests were significant for AAV-GFP-deafened animals at DOS (*p* = 0.0007) and for AAV-FoxP2-deafened animals at D14, D25, D60, and DOS (*p* = 0.0006, *p* = 0.0002, *p* < 0.0001, and *p* < 0.0001, respectively; Fig. 3*B*). A *post hoc* Sidak’s multiple comparisons test showed that at no time point did groups differ from one another. Values for GFP-Deaf and FoxP2-Deaf groups showed the greatest separation from each other at D14 (mean ± SEM: GFP, 0.870 ± 0.092; FoxP2, 0.644 ± 0.047; *p* = 0.265) and D25 (mean ± SEM: GFP, 0.840 ± 0.059; FoxP2, 0.653 ± 0.110; *p* = 0.302); *p* values at all other time points were *p* > 0.8.

**Figure 4.**
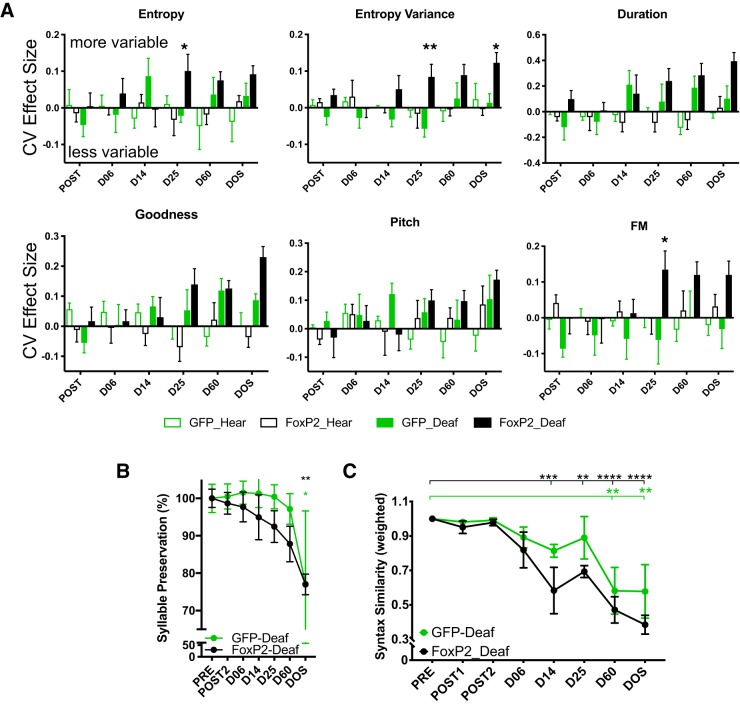
Spectral variability and sequencing are affected by FoxP2 overexpression in deaf birds. ***A***, Vocal variability increased more rapidly in deafened AAV-FoxP2 birds (solid black bars) than in deafened AAV-GFP birds (solid green bars) in most song features analyzed (e.g., entropy, entropy variance, duration, pitch goodness, FM). Positive values indicate an increase in the CV of each feature relative to PRE; negative values reflect lower variability than observed in PRE. ***B***, Syllable omission occurs faster in FoxP2-deafened animals than in GFP-deafened animals. ***C***, Syntax similarity (syllable sequencing; normalized to PRE) is disrupted in FoxP2-deafened animals by 14 d following deafening (*p < 0.05, **p < 0.01, ***p < 0.001, ****p < 0.0001.) *Figure Contributions:* Nancy Day performed the experiments and analyzed the data.

Early-onset song deterioration in adult males overexpressing FoxP2 without auditory feedback could be the result of spectral degradation and/or changes in song sequencing. To distinguish between these, we quantified the effect of deafening on the CV of acoustic features in all groups. Deafened animals overexpressing FoxP2 showed greater variability in three spectral features at earlier time points relative to deafened GFP animals (Fig. 4*A*). At D25, entropy (*p* = 0.025), entropy variance (*p* = 0.004), and FM (*p* = 0.04) were more variable in FoxP2-Deaf birds compared to GFP-Deaf birds (two-way ANOVA with Sidak’s test for multiple comparisons). Additionally, entropy variance was significantly more variable on DOS (*p* = 0.04) in FoxP2-deaf birds. However, GFP-Deaf birds, compared to FoxP2-Deaf birds, did not show a significant increase in the variability of any spectral feature at any time point. No statistically significant differences were observed for any spectral feature at any time point in the two groups of hearing animals (a two-way ANOVA was performed for each spectral feature between hearing groups over time; none were significant). Finally, we examined the presence/absence of each syllable following deafening and the sequencing of song syllables. We observed that deafened AAV-FoxP2 animals dropped syllables from their motifs more rapidly than AAV-GFP-deafened animals (Fig. 4*B*); however, the percentage of dropped syllables was not significant between groups (two-way ANOVA: group: *F*_(1,61)_ = 3.017, MS = 0.050, *p* = 0.087; time: *F*_(6,61)_ = 4.39, MS = 0.072, *p* = 0.0010; interaction: *F*_(6,61)_ = 0.27, MS = 0.004, *p* = 0.949). Over the course of recording, Sidak’s *post hoc* test showed that both GFP-deaf and FoxP2-deaf animals had significantly fewer syllables at PRE versus DOS (*p* = 0.045 and *p* = 0.0073, respectively). Lasting syntactical changes were present as early as D14 in AAV-FoxP2 animals compared to the later onset of these changes at D60 in AAV-GFP animals (Fig. 4*C*). Together, these results indicate that a combination of spectral and sequencing alterations underlie the acceleration of deafening-induced song deterioration in animals overexpressing FoxP2 in area X.

### Female-directed song is more variable following FoxP2 overexpression

**Figure 5.**
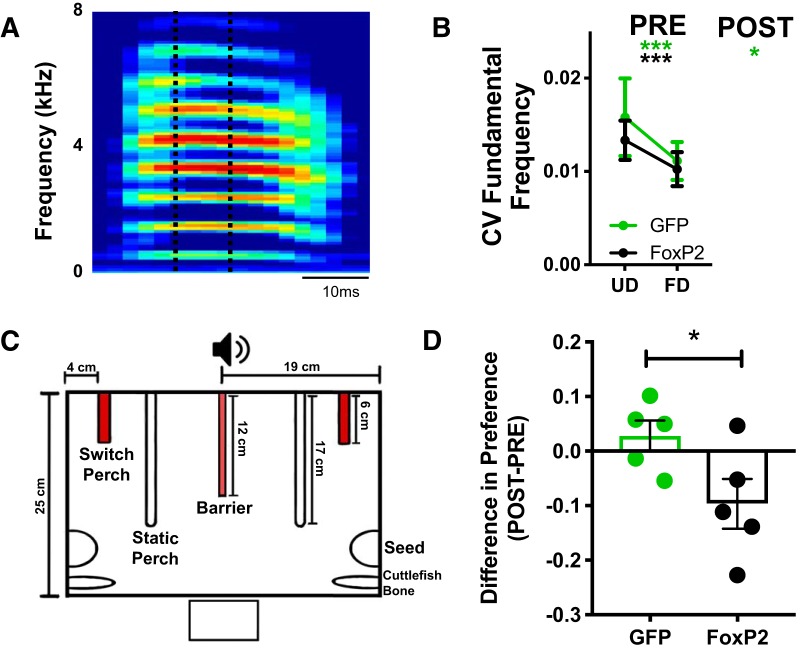
Female conspecifics perceive alterations in social context-dependent song variability. ***A***, Exemplar syllable with a harmonic element/stack. Only the “flat” component of the syllable (indicated by dotted lines) was analyzed to determine the CV of the FF. ***B***, Before AAV injections, syllables are performed with less rendition-to-rendition variability during female-directed song compared to UD in both GFP and FoxP2 groups (Wilcoxon matched-pairs signed-rank test, one-tailed; AAV-GFP: *p* = 0.0002, *n* = 12 syllables, AAV-FoxP2: *p* = 0.0001, *n* = 13 syllables). Following AAV injection, the CV of FF is significantly lower for female-directed syllables in GFP-injected zebra finches (Wilcoxon matched-pairs signed-rank test, one-tailed, *p* = 0.017, *n* = 12 syllables), but not in FoxP2-injected animals (Wilcoxon matched-pairs signed-rank test, one-tailed, *p* = 0.064, *n* = 13 syllables; *p < 0.05, ***p < 0.001). ***C***, “Bird’s eye view” schematic of the testing arena for assaying female preference. ***D***, Female preference for FD song is reduced (Preference_POST_ – Preference_PRE_) following FoxP2 overexpression compared to songs following GFP overexpression (two-tailed *t* test, *p* = 0.047, *t* = 2.34, df = 8, *n* = 5 male birds per group). *Figure Contributions:* Taylor Hobbs designed the female preference testing area. Taylor Hobbs and Nancy Day performed the female preference experiments and analyzed the data.

Syllables with harmonic elements are sung with less rendition-to-rendition variability during female-directed song than UD (Kao et al., 2005). Knock-down of FoxP2 within area X of adult zebra finches abolishes this social context-dependent change in vocal variability, as measured by the CV of the FF (Murugan et al., 2013). We calculated the CV of FF in the harmonic elements of syllables in hearing birds to determine if FoxP2 overexpression alters rendition-to-rendition variability in female-directed song (Fig. 5*A*). As expected, before overexpression of FoxP2 or GFP, harmonic elements were performed with a significantly lower CV during female-directed song compared to UD [AAV-GFP: UD Pre vs FD Pre, *p* = 0.0002, *n* = 12 syllables (six birds), one-tailed Wilcoxon matched-pairs signed-rank test; AAV-FoxP2: UD Pre vs FD Pre, *p* = 0.0001, *n* = 13 syllables (seven birds), one-tailed Wilcoxon matched-pairs signed-rank test]. However, after FoxP2 overexpression, the CV of harmonic elements in FD song was no longer significantly different from UD renditions (*p* = 0.064, one-tailed Wilcoxon matched-pairs signed-rank test; Fig. 5*B*). AAV-GFP birds continued to perform FD song with lower variability than UD song (one-tailed Wilcoxon matched-pairs signed-rank test, *p* = 0.0002, *n* = 12 syllables from six birds). We compared the mean number of introductory notes, the mean bout duration, and mean motif duration in both PRE and POST songs (UD and FD). We did not find any differences in these metrics following virus injections (data not shown).

Multiple measures of mRNA expression reveal that area X *FoxP2* levels are lower in adult males following UD than following production of highly-stereotyped female-directed song (Teramitsu and White, 2006). One prediction based on these observations was that preventing FoxP2 downregulation by overexpression may result in songs with lower rendition-to-rendition variability than is typically present in UD. In contrast to this idea, there were no song features in which FoxP2 overexpression reduced vocal variability (Fig. 4*A*). The CV of the FF of harmonic elements within syllables did not change in UD following AAV-GFP (two-tailed Wilcoxon matched-pairs signed-rank test, *p* = 0.083, *n* = 11 syllables) or AAV-FoxP2 (two-tailed Wilcoxon matched-pairs signed-rank test, *p* = 0.094, *n* = 13 syllables) injection.

### FoxP2 overexpression tempers females’ preference for female-directed song

Female preference for male song is inversely correlated with song variability (Woolley and Doupe, 2008; Chen et al., 2017; Heston et al., 2018). To determine if increased variability of FD song induced by FoxP2 overexpression was perceived by conspecifics, and thus of potential ethological relevance, we tested whether female zebra finches altered their behavior in response to more stereotyped (AAV-FoxP2 PRE FD) or variable (AAV-FoxP2 POST FD) songs. We used a perch-hop paradigm (Fig. 5*C*; see Materials and Methods) to measure sexually-naive females’ preferences for songs performed under different social (UD vs FD) and viral (PRE vs POST; GFP vs FoxP2) conditions. We accounted for each female’s bias for activating a specific perch by calculating an effect size for perch preference ([Perch 1 – Perch 2]/[Perch 1 + Perch 2]) when no playbacks were presented (silence) versus when playbacks of either FD or UD song were paired with perch activations. (Notably, FD song playbacks were always paired with the lesser PP during the silence testing period.) To obtain a “preference score” (Fig. 5*D*), the effect size of the silence testing period was subtracted from the effect size of the playback testing period (preference scores > 0 indicate a preference for FD song). The median preference score from at least five females was calculated between subjects for each male.

As expected, females demonstrated a preference for FD song compared to UD (preference score > 0; *p* = 0.0006, two-tailed one-sample *t* test, *n* = 10 male birds). Overall, we found that while females still preferred FD song to UD song sung by AAV-FoxP2-injected males, their preference for those songs was diminished relative to songs sung before AAV injection (*p* = 0.051, one-tailed paired *t* test, *n* = 5 male birds). The preference for FD song following AAV-GFP surgery was unchanged (*p* = 0.182, one-tailed paired *t* test, *n* = 5 male birds).

## Discussion

The transcription factor FoxP2 is critical to the proper development of learned vocalizations used for social communication in both humans and zebra finch songbirds. Here, we provide novel evidence that the maintenance of learned vocalizations in adulthood relies on auditory-dependent regulation of striatopallidal FoxP2. In juvenile finches, the shared behavioral outcomes that follow FoxP2 overexpression or knock-down suggest that song learning is dependent on behavior-driven regulation of FoxP2 in the striatopallidal song-dedicated nucleus area X; having too much, or too little, results in similar deficits (Haesler et al., 2007; Heston and White, 2015; Burkett et al., 2018). Behavior-driven FoxP2 regulation also occurs in adults (Teramitsu and White, 2006; Miller et al., 2008; Hilliard et al., 2012a; Shi et al., 2013; Thompson et al., 2013), which motivated us to test for a possible role forFoxP2in the maintenance of learned vocalizations. We confirmed that, in hearing birds, area X FoxP2 levels affect the precision of courtship song (Murugan et al., 2013). Going further, our data suggest that the auditory feedback required to maintain adult song may do so, in part, through regulation of area X FoxP2 levels. Together, these findings indicate that appropriate behavioral regulation of FoxP2 is not only critical for juveniles who are in the process of song learning, but also for adult animals who require ongoing auditory feedback to properly maintain their song.

An experimental strength offered by adult zebra finch song is its robustness, characterized by marked stability across song renditions throughout the lifespan. This provides an easily quantifiable behavior for assessing the effects of mechanistic interventions. Such behavioral stability may reflect a fixed nature of its biological underpinnings. Indeed, a historical assumption was that AFP song control nuclei were unnecessary for adult song maintenance since limited-to-no changes in song were detected following lesions of these areas in adults. This was in marked contrast to the profound effects on learning observed after lesioning these regions in juveniles or the dramatic loss in learned vocal output that follows lesions of nuclei in the vocal motor pathway at any age (Bottjer et al., 1984; Scharff and Nottebohm, 1991).

Subsequent landmark experiments unveiled an ongoing role for the AFP in adult song maintenance by combining two interventions, i.e., by assessing changes to song following both lesioning and deafening (Brainard and Doupe, 2000). In birds with an intact AFP, deafening resulted in song degradation, as previously shown (Nordeen and Nordeen, 1992, 2010; Brainard and Doupe, 2001; Horita et al., 2008). Strikingly, lesions of the AFP prevented deafening-induced song deterioration (Brainard and Doupe, 2000; Kojima et al., 2013). Thus, this “double-insult” methodology unveiled the normal role of the AFP in song maintenance by actively generating vocal variability in adults (Woolley and Kao, 2015). By analogy, here we tested the role of FoxP2 in adult maintenance by introducing a genetic “lesion,” i.e., by blocking natural behavior-linked FoxP2 cycling in area X through viral-driven overexpression. Similar to lesions of the AFP, we detected fairly subtle effects of our genetic insult in hearing birds, consistent with the robust stability of adult song. Likewise, disruptions to cortico-striatal circuits in humans and rodent models induce more prominent deficits during learning than during execution of well-learned skills (Graybiel, 2008; Kawai et al., 2015). In striking contrast, when the genetic insult was paired with deafening, it accelerated song decrystallization, revealing a role for behaviorally-regulated FoxP2 expression in ongoing song maintenance.

It is important to note that overexpression of FoxP2 does not simply recapitulate the effect of lesioning area X in adult finches. While both chemical and genetic insults to area X result in few changes in the songs of hearing birds, the experimental outcomes diverge in deafened animals. Chemical lesions of area X prevented deafening-induced song deterioration (Kojima et al., 2013) whereas our genetic manipulation accelerated song degradation. These results extend our prior observation that hearing regulates area X FoxP2 expression during sensorimotor learning (Teramitsu et al., 2010). In both deafened and hearing juvenile finches, FoxP2 was downregulated following 2 h of UD singing, indicating that FoxP2 expression is primarily regulated by motor activity. However, FoxP2 expression and amount of singing were not correlated in deafened juveniles as they were in hearing juveniles. This suggests that while motor behavior is sufficient to decrease area X FoxP2 levels, auditory feedback is necessary to properly calibrate its expression. Additionally, a notable trend in the deafened-FoxP2 injected animals was an increase in FoxP2 expression relative to other groups, despite singing similar amounts of song before sacrifice (Fig. 1*F*). This suggests that the lack of auditory feedback was insufficient to proportionally lower FoxP2 as observed in the FoxP2-hearing animals. Molecular regulators of FoxP2 such as POU3F2 (Atkinson et al., 2018), miR-9 and miR-140-5p (Shi et al., 2013) have been identified. Thus, it will be important to determine how sensory feedback affects the regulation of these molecules and, in turn, FoxP2 in the coordination of complex motor tasks.

The hastening of deafening-induced song deterioration and increase in phonological and sequencing variability following FoxP2 overexpression, suggests that (1) auditory feedback is critical for the proper function of FoxP2 to precisely control mature vocalizations and (2) dysregulated FoxP2 increases song variability. Indeed, similar to knock-down of FoxP2 in area X of adult zebra finches (Murugan et al., 2013), we observed an increase in the acoustic variability of female-directed song, indicating that FoxP2 may mediate an adult’s ability to generate appropriate behavioral responses to salient social cues. This is consistent with the result that either overexpression or knock-down of FoxP2 impairs song copying in juvenile finches (Haesler et al., 2007; Heston and White, 2015). Together, these convergent findings suggest that interfering either by overexpression or by knock-down of FoxP2 produces similar behavioral outcomes in adults, as in juveniles. Our data also strengthen a model in which self-regulation of FoxP2 by sensory and motor cues enable song variability that is necessary for ongoing refinement of learned vocalizations.

Social-context driven changes to song variability have been associated with dopamine modulation in area X (Sasaki et al., 2006; Leblois et al., 2010; Leblois and Perkel, 2012; Murugan et al., 2013). In particular, the marked stability of female-directed song depends on activation of D1 receptors (Leblois et al., 2010). We found that *FoxP2* expression positively correlates with *D1R* expression (Fig. 1*G*) and increases the rendition-to-rendition variability of the FF of syllables containing harmonic stacks. In our study, dopamine receptor transcript levels were assessed before the onset of singing and in the absence of any females. Thus, changes in dopamine marker levels may not correlate with physiologic changes that occur when birds are actively singing or when in the presence of females. This difference in experimental protocol may account for our findings relative to previous reports that show low acoustic variability following D1R receptor antagonism in area X (Leblois et al., 2010).

The mechanisms that reinforce optimal motor patterns within cortico-basal ganglia circuits increasingly implicate a critical role for dopamine (Schultz et al., 1997; Graybiel, 2008; Murugan et al., 2013; Gadagkar et al., 2016; Hoffmann et al., 2016; Xiao et al., 2018). FoxP2 is linked to intracellular dopaminergic signaling to influence vocal variability (Vernes et al., 2011; Murugan et al., 2013), but it remains untested as to whether mechanisms that alter signal propagation in the AFP following FoxP2 knock-down are the same as those that may accompany FoxP2 overexpression. Elucidating the interaction between FoxP2 and dopaminergic signaling, particularly given that the ventral tegmental area (VTA) receives afferents from multiple auditory regions (Mandelblat-Cerf et al., 2014) and dopamine signaling encodes performance errors during singing (Gadagkar et al., 2016), will be essential in understanding its ongoing role in song maintenance and modulation during social communication. Additional experiments will also be necessary to determine if afferents from HVC, a critical conveyer of auditory input to the AFP (Roy and Mooney, 2007; Gale and Perkel, 2010), calibrate expression of area X FoxP2 despite evidence that HVC does not transmit error-related signals (Hessler and Doupe, 1999; Kozhevnikov and Fee, 2007) or receive auditory signals (Hamaguchi et al., 2014) during singing.

Our study provides insight into how FoxP2 may influence social communication between conspecifics and identifies FoxP2 as necessary for the execution of precise motor behaviors. We used females to demonstrate that the increase in vocal variability following FoxP2 overexpression has functional consequences. Females prefer stereotyped song with low rendition-to-rendition variability (Woolley and Doupe, 2008; Dunning et al., 2014; Chen et al., 2017). The decrease in female preference for FD song following FoxP2 overexpression is consistent with the observed increase in vocal variability in those songs. Using females to identify whether experimenter-induced changes to male song promote or impede song quality can thus tease out ethologically-relevant manipulations to song.

Within neural circuits that control behavior, the FoxP2 transcription factor can coordinate the activation or repression of hundreds to thousands of genes, affecting a variety of molecular mechanisms (Vernes et al., 2011; Hilliard et al., 2012a; Chen et al., 2016). Gene co-expression patterns within area X shift across the critical period from song learning to song maintenance (Burkett et al., 2018), suggesting that individual genes, including FoxP2, can differentially contribute to a variety of behaviors, including both learning in juveniles and maintenance in adults. Although no differences in gene expression of transcription factors have been identified in the cortical song motor pathway following deafening (Mori and Wada, 2015), we predict that auditory deprivation will influence gene expression patterns in the avian striatum. Thus, in the future, it will be necessary to identify how FoxP2 overexpression in the presence or absence of auditory feedback alters gene co-expression. Such experiments may illuminate how FoxP2 orchestrates the molecular microcircuitry necessary for song maintenance, and, by extension, human speech.

## Synthesis

Reviewing Editor: Darcy Kelley, Columbia University Decisions are customarily a result of the Reviewing Editor and the peer reviewers coming together and discussing their recommendations until a consensus is reached. When revisions are invited, a fact-based synthesis statement explaining their decision and outlining what is needed to prepare a revision will be listed below. The following reviewer(s) agreed to reveal their identity: NONE. Note: If this manuscript was transferred from JNeurosci and a decision was made to accept the manuscript without peer review, a brief statement to this effect will instead be what is listed below.

In vocal motor learning, sounds used for social communication are shaped by auditory experience. Vocal learning is quite rare but characterizes both speech in humans and song in oscine birds. Shared features of vocal learning - including a well defined critical period and a role for the transcription factor FoxP2 - have made zebra finches in particular, a useful model for understanding how CNS plasticity supports learning during development and the role FoxP2 may play in this process. Plasticity is not confined to development: deafening in adulthood degrades vocal production. Results described in this paper are of interest because they demonstrate that over-expression of FoxP2 can exacerbate vocal degradation sufficiently to affect a female's preference for a song. While the paper does not nail down exactly how this works, the finding is likely to be of considerable interest in its present form and the authors have carefully revised the manuscript in response to previous reviews (e.g. new Figure 5D).

A few suggestions:

Lines 3 - 4 “The neural underpinnings of speech can be investigated in

songbirds, because, like speech, birdsong is learned ...” There are many parallels between the two but it is still not clear that bird song actually “illuminates human speech” per se. Suggest simply beginning with “Like speech, birdsong is ...”

Lines 13 -14 Suggest: “...overexpressed FoxP2 in both hearing and deafened adult zebra finches and assessed effects on songs sung while alone versus songs directed to females. In intact birds singing alone, no changes were detected in songs of males expressing FoxP2 or a GFP control in Area X.”

Lines 16 - 17 Suggest: “In contrast, songs of males overexpressing FoxP2, became more variable and were less preferred by females, unlike GFP-expressing control males:”

Line 71. Suggest starting new paragraph with: “In adults, knockdown of FoxP2 .... and going directly to ”To reveal 76 whether FoxP2 participates in active song maintenance, we prevented behavior-driven i.e. delete “The above findings motivate...” sentence.
